# AGSK-Net: Adaptive Geometry-Aware Stereo-KANformer Network for Global and Local Unsupervised Stereo Matching

**DOI:** 10.3390/s25185905

**Published:** 2025-09-21

**Authors:** Qianglong Feng, Xiaofeng Wang, Zhenglin Lu, Haiyu Wang, Tingfeng Qi, Tianyi Zhang

**Affiliations:** School of Mathematical and Physical Sciences, Chongqing University of Science and Technology, Chongqing 401331, China; 2023211020@cqust.edu.cn (Q.F.); 2023211001@cqust.edu.cn (Z.L.); 2023211019@cqust.edu.cn (H.W.); 2024211017@cqust.edu.cn (T.Q.); 2024211019@cqust.edu.cn (T.Z.)

**Keywords:** unsupervised stereo matching, epipolar geometry prior, adaptive fusion, KAN, swin transformer

## Abstract

The performance of unsupervised stereo matching in complex regions such as weak textures and occlusions is constrained by the inherently local receptive fields of convolutional neural networks (CNNs), the absence of geometric priors, and the limited expressiveness of MLP in conventional ViTs. To address these problems, we propose an Adaptive Geometry-aware Stereo-KANformer Network (AGSK-Net) for unsupervised stereo matching. Firstly, to resolve the conflict between the isotropic nature of traditional ViT and the epipolar geometry priors in stereo matching, we propose Adaptive Geometry-aware Multi-head Self-Attention (AG-MSA), which embeds epipolar priors via an adaptive hybrid structure of geometric modulation and penalty, enabling geometry-aware global context modeling. Secondly, we design Spatial Group-Rational KAN (SGR-KAN), which integrates the nonlinear capability of rational functions with the spatial awareness of deep convolutions, replacing the MLP with flexible, learnable rational functions to enhance the nonlinear expression ability of complex regions. Finally, we propose a Dynamic Candidate Gated Fusion (DCGF) module that employs dynamic dual-candidate states and spatially aware pre-enhancement to adaptively fuse global and local features across scales. Experiments demonstrate that AGSK-Net achieves state-of-the-art accuracy and generalizability on Scene Flow, KITTI 2012/2015, and Middlebury 2021.

## 1. Introduction

Stereo matching is one of the core tasks in computer vision and plays a critical role in 3D scene reconstruction. This 3D perception capability makes it a fundamental component in various application domains, such as autonomous driving [1], 3D reconstruction [2], and robotic navigation [3].

In recent years, deep learning has made significant progress in the field of computer vision. Currently, deep learning-based stereo matching can be broadly categorized into supervised learning [4,5,6] and unsupervised learning [7,8,9,10,11,12]. Supervised learning approaches heavily rely on annotated disparity values, and obtaining large-scale, high-quality depth labels is costly. Moreover, the generalization ability in new scenes is limited. Unsupervised stereo matching eliminates the reliance on ground-truth disparity labels, enabling training on large unlabeled datasets and improving cross-domain generalization and the feasibility of engineering deployment. Moreover, recent studies [13,14] have indicated that the unsupervised paradigm holds significant potential for development in handling mismatched annotation modalities, improving generalization, and enhancing robustness in real-world applications, making it a key direction for future research.

However, existing unsupervised approaches [7,8,9,10,11,12] struggle in complex regions requiring global context, such as weak textures and occlusions, mainly because they rely on convolutions with limited receptive fields constrained by their local nature.

To address this, Vision Transformers (ViTs) [15] have been introduced for their exceptional ability to model long-range dependencies [16,17,18,19]. However, their direct application to stereo matching reveals three critical, unaddressed limitations. Firstly, the isotropic nature of the standard self-attention mechanism treats displacements in all directions equally, whereas the search space for stereo image matching is typically restricted by horizontal epipolar geometric priors. This may lead to the ingestion of redundant contextual information, interfering with the critical horizontal matching procedure. Existing geometry-aware approaches [20,21,22,23] mainly apply epipolar constraints during cross-view matching, leaving the issue of geometric consistency in feature extraction largely unaddressed. Secondly, the Multi-layer Perceptron (MLP) [24], widely used in Transformers for nonlinear modeling, relies on fixed, nonlearnable activation functions and simple linear transformations. This design limits its expressive capacity and makes it difficult to capture the nonlinear relationships in complex regions of stereo matching. In contrast, the Kolmogorov–Arnold Network (KAN) [25] enhances nonlinear expressiveness through flexible and learnable univariate functions, showing strong potential for dense tasks. However, the combination of KAN with ViTs has not yet been applied to dense tasks. Existing studies have primarily focused on sparse target scenarios, such as categories [26] and objects [27]. Finally, effective stereo matching requires not only global context for geometry-aware reasoning but also fine-grained local details for accurate complex detail recognition. Therefore, fusing global and local features becomes a critical step in stereo matching. Traditional fusion approaches [16,17,18,19] typically rely on simple stacking, weighting, or attention guidance. However, these methods often introduce redundant information or show attention bias toward a specific modality, which can lead to the loss of critical information and compromise overall model performance.

To systematically address the above challenges, we build upon the Swin Transformer [18] and develop the Adaptive Geometry-aware Stereo-KANformer Network (AGSK-Net). The core idea is to enhance global reasoning while preserving fine-grained local details under the guidance of epipolar geometry. Specifically, we introduce three key innovations in the Stereo-KANformer backbone. First, the Adaptive Geometry-aware Multi-head Self-Attention (AG-MSA) embeds epipolar priors through Adaptive Geometry-enhanced Relative Positioning (AGRP), which imposes anisotropic attention biases. AGRP forms a soft epipolar band that tolerates slight vertical perturbations while suppressing nonepipolar responses, thereby reshaping spatial prior integration and improving geometric consistency during feature extraction. Second, the Spatial Group-Rational KAN (SGR-KAN), inspired by the KAN paradigm [25,28], replaces conventional MLPs with flexible group-rational functions directly applied to 2D feature maps. This design preserves spatial topology and enables localized channel–spatial nonlinear modeling, significantly boosting expressive power in complex regions. Third, the Dynamic Candidate Gated Fusion (DCGF) module integrates CNN-derived local details with geometry-enhanced global features from the Stereo-KANformer. It introduces a dual-candidate gating mechanism combined with coordinate attention, which dynamically selects and balances complementary feature streams. As a result, DCGF mitigates redundancy, preserves critical details, and achieves fine-grained global–local fusion. Together, these components form an end-to-end unsupervised stereo matching framework that improves accuracy and robustness, particularly in challenging regions with weak textures, occlusions, or other ill-posed conditions. Our contributions are threefold:

- We propose a novel Adaptive Geometry-aware Multi-head Self-Attention (AG-MSA) for unsupervised stereo matching. Our approach introduces an AGRP attention bias modulation framework, which encodes epipolar geometry priors through a hybrid design of geometric functions for adaptive modulation and penalty, replacing conventional isotropic or simple additive biases and thereby enhancing the geometric consistency and global inference capability.
- We design a Spatial Group-Rational KAN (SGR-KAN) for unsupervised stereo matching. By integrating the powerful nonlinear expressive capability of rational functions with the spatial perception ability of deep convolution, flexible and learnable Group-Rational KAN are directly applied on 2D feature maps to replace MLP. This enables channel-wise and spatial grouped modeling, thereby explicitly preserving spatial structure and enhancing the nonlinear expressive capability in complex regions.
- We propose a Dynamic Candidate Gated Fusion (DCGF) module for global and local unsupervised stereo matching. The module constructs a novel dynamic dual-candidate state mechanism and coordinate attention mechanism enhanced with spatial information and adaptively arbitrates between different fusion strategies based on feature content, ensuring a more effective and complementary fuse of information from the CNN and Stereo-KANformer backbones.

Extensive experiments on standard benchmarks, including Scene Flow, KITTI, and Middlebury, demonstrate that AGSK-Net achieves state-of-the-art performance, particularly in challenging ill-posed regions, validating the effectiveness of our integrated design.

## 2. Related Work

### 2.1. CNN-Based Unsupervised Stereo Matching

Convolutional Neural Networks (CNNs) have long been the cornerstone of stereo matching. Early unsupervised approaches [7,8,9,10] establish a foundational paradigm based on view synthesis and left–right disparity consistency. In this setting, a network is trained to reconstruct a target view from a source view using the predicted disparity map. However, the accuracy of the resulting disparity maps remains limited. SMAR-Net [11] employs stacked stereo image pairs to predict disparity, but this strategy faces challenges in large disparity variations and complex scenes. As one of the most popular unsupervised models, PASMNet [12] adopts cascaded cost aggregation and disparity attention mechanisms, effectively addressing the issue of fixed disparity ranges. However, despite significant progress, existing unsupervised stereo matching approaches [7,8,9,10,11,12] still struggle in complex regions such as occlusions and weak textures. This highlights the need for models that can move beyond purely local operations and incorporate global contextual reasoning.

### 2.2. Transformer-BasedStereo Matching

To overcome the locality limitations of CNNs, Vision Transformers (ViTs) [15] and their variants have been introduced to the stereo matching field. STTR [16] is a pioneering work that frames stereo matching as a sequence-to-sequence problem, leveraging the global self-attention mechanism of Transformers to directly find correspondences. Following this, architectures based on the more efficient Swin Transformer [18], such as those proposed by Jia et al. [17] and STFC-Net [19], demonstrate strong performance by combining hierarchical feature representation with shifted-window attention. These models confirm the immense potential of Transformers in capturing global context and achieving impressive cross-domain generalization. However, these works typically adopt the standard, geometry-agnostic self-attention mechanism, failing to explicitly incorporate the fundamental epipolar geometric prior of stereo vision into their core design, which may limit the ability in modeling geometric consistency.

### 2.3. Epipolar Geometry in Stereo and Multi-View Methods

Recent works explore epipolar geometry guidance in both stereo and multi-view settings. H-Net [20] introduces mutual epipolar attention within an unsupervised stereo framework to emphasize correspondences on the same epipolar line and employs optimal transport to suppress outliers. MVSTER [23] extends epipolar guidance into the multi-view stereo (MVS) domain by proposing an epipolar Transformer that constrains cross-view attention along epipolar lines. It further introduces a detachable monocular-depth branch and cascade strategies to build data-dependent 3D associations, thereby improving reconstruction efficiency and completeness. Inspired by MVSTER, EI-MVSNet [21] introduces epipolar guidance volume construction and interval-aware depth labeling to refine the candidate depth intervals and cost volume aggregation. The geometry-enhanced attentive approach [22] further exploits geometric cues to produce multi-view consistent features under challenging illumination and low-texture scenarios. While these methods [20,21,22,23] exploit epipolar priors at the cross-view matching or cost aggregation stages, they seldom integrate such priors into single-view feature extraction. Most intra-view streams still rely on standard isotropic backbones (CNNs or ViTs), so redundant nonepipolar context may be encoded before matching, which limits the potential benefits of geometry guidance.

### 2.4. Kolmogorov–Arnold Networks

Traditional neural networks, including MLP within Transformers, rely on fixed and nonlearnable activation functions and linear transformations, which can limit their capability to model highly complex, nonlinear functions. Recently, Kolmogorov–Arnold Networks (KANs) [25], derived from the superposition theorem, enhance expressivity and interpretability by replacing node-based MLP with edge-wise learnable univariate functions. Recent studies demonstrate their effectiveness in computer vision. U-KAN [27] integrates KAN layers into U-Net bottlenecks, achieving higher segmentation accuracy with reduced parameters and FLOPs. Extensions such as KM-UNet [29] and MM-UKAN++ [30] further combine KAN with state-space models or multiscale fusion, showing consistent gains on medical image benchmarks. Recently, Yang and Wang proposed the Kolmogorov–Arnold Transformer (KAT) [28], which substitutes Transformer feed-forward layers with variant GR-KAN and introduces rational function bases and grouped parameter sharing to improve scalability on large-scale datasets. However, it often requires flattening features and does not inherently handle the 2D spatial structure of images, which discards crucial topological information. As a result, valuable topological relationships and relative positional cues are often lost, making it difficult for KANs to fully exploit geometric structures in dense prediction tasks. These limitations suggest that further progress depends on integrating KANs into architectures that explicitly preserve spatial awareness, for example, by combining them with convolutional backbones or attention mechanisms designed for structured 2D data.

## 3. Materials and Methods

To address these limitations, we propose the Adaptive Geometry-aware Stereo-KANformer Network (AGSK-Net), which integrates geometry-aware attention, enhanced nonlinear modeling, and adaptive global and local fusion. Therefore, we first design a novel Stereo-KANformer feature extraction architecture, which employs Adaptive Geometry-aware Multi-head Self-Attention (AG-MSA) and Spatial Group-Rational KAN (SGR-KAN), systematically addressing the key challenges of insufficient global reasoning capability, lack of geometric prior information, and limited nonlinear expressive capacity. In addition, we further propose a Coordinate Attention-enhanced Dynamic Gating Fusion module (DCGF) to achieve efficient and adaptive fusion of multi-scale global and local features. This section will elaborate on the overall architecture of AGSK-Net and the specific design of the aforementioned core modules.

### 3.1. Overview

**Figure 1:**
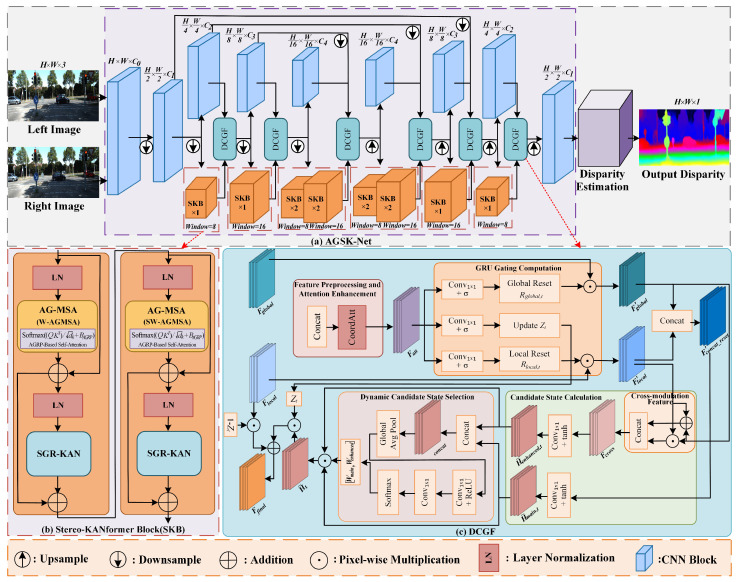
The overall architecture of the proposed approach. $C_{0}$, $C_{1}$, …, $C_{4}$ denote the feature maps at different hierarchical stages of the network, where deeper levels correspond to lower spatial resolutions and larger channel dimensions. (**a**) Overall architecture of AGSK-Net. The network combines CNN and Stereo-KANformer to extract multi-scale local and global features from left and right images, achieves adaptive fusion through the DCGF module, and ultimately generates fused feature maps for disparity estimation. (**b**) The structure of Stereo-KANformer Block. It utilizes AG-MSA and SGR-KAN to extract geometry-aware global contextual information and enhance nonlinear expressive capability in complex regions. (**c**) The structure of DCGF module. The module adaptively fuses global and local features at different scales to generate more informative fused features.

This paper proposes an Adaptive Geometry-aware Stereo-KANformer Network (AGSK-Net) for dense unsupervised stereo matching. The overall architecture is illustrated in Figure 1a. Given a pair of stereo images from the left and right views, $I_{L},I_{R}\in R^{H\;\times \;W\;\times \;3}$ as input, multi-scale features of the left and right views are first extracted through an encoder–decoder backbone network based on CNN and Stereo-KANformer. The backbone network utilizes convolutional layers to extract local detail features. Meanwhile, the proposed Stereo-KANformer Block (SKB) is introduced into the deeper layers and bottleneck layers of the encoder and decoder networks to capture geometry-aware global contextual information, enhancing the nonlinear expressive capability in complex regions of the spatial structure. Then, to fully fuse the fine-grained local detail features $F_{\mathrm{local}}$ extracted by the CNN with the geometry-aware global context features $F_{\mathrm{global}}$ captured by SKB, we employ the further designed DCGF to adaptively fuse these two heterogeneous features at multiple scales, generating a more informative and discriminative fused feature $F_{\mathrm{final}}$. Finally, the fused features are used to estimate the disparity map.

### 3.2. Stereo-KANformer

The Swin Transformer architecture typically overlooks a fundamental anisotropic geometric prior in stereo matching. Its isotropic relative position bias mechanism assigns equal weights to relative displacements in all directions, ignoring the geometric characteristics of binocular stereo matching. The neglect of critical geometric constraints leads to redundant context learning, which introduces noise in occluded or textureless regions and ultimately degrades matching accuracy. Additionally, Swin Transformer architecture often relies on MLP [24] to achieve nonlinear feature mapping. This limits its feature representation capability, particularly when handling complex geometric correspondences and subtle texture variations in stereo matching. Based on this, this paper introduces anisotropic epipolar geometric priors into the multi-head self-attention mechanism to achieve effective geometric perception. Leveraging the powerful nonlinear feature representation capability of KAN, we propose the Stereo-KANformer architecture, a KAN-based geometry-aware Swin Transformer for unsupervised stereo matching. By guiding feature learning with epipolar geometric priors, the model enhances geometric consistency along the disparity dimension, thereby capturing global contextual information and enhancing its capacity for modeling nonlinear correspondences in complex scenes.

Specifically, an Adaptive Geometry-enhanced Multi-head Self Attention (AG-MSA) mechanism tailored for unsupervised stereo matching is first designed, drawing inspiration from the efficient window-based architecture of Swin Transformer [18]. The proposed Window-based Adaptive Geometry-enhanced Multi-head Self Attention (W-AGMSA) and Shifted Window-based Adaptive Geometry-enhanced Multi-head Self Attention (SW-AGMSA) are applied consecutively to enhance geometric perception, which enables the extraction of global contextual information within each window. Secondly, Spatial Group-Rational KAN (SGR-KAN) is directly applied on the 2D feature maps to replace the MLP module in the traditional Swin Transformer, improving nonlinear expressive capability in complex regions, preserving the spatial characteristics of features, and making it more suitable for the dense unsupervised stereo matching.

#### 3.2.1. Stereo-KANformer Block (SKB)

A standard Stereo-KANformer Block (SKB), as shown in Figure 1b, introduces geometric priors and the powerful nonlinear capability of KAN through four stages. A key design principle of the SKB is the consecutive application of two complementary attention modules, Window-based AG-MSA (W-AGMSA) and Shifted-Window-based AG-MSA (SW-AGMSA), to efficiently model both local and global dependencies. Their distinct roles are as follows:

W-AGMSA performs self-attention strictly within nonoverlapping local windows. This approach is highly efficient, as it limits the quadratic complexity of the attention computation to the size of each window. However, this efficiency comes at the cost of lacking information flow between windows, confining the receptive field of this step. SW-AGMSA is designed specifically to overcome this limitation. By applying a cyclic shift to the feature map before partitioning it into windows, SW-AGMSA creates new window configurations that bridge the boundaries of the previous W-AGMSA step. This crucial operation enables cross-window connections, allowing information to be exchanged across the entire feature map.

The consecutive pairing of a W-AGMSA stage with an SW-AGMSA stage in successive SKBs is therefore essential. This design allows the Stereo-KANformer backbone to build a global receptive field and capture long-range dependencies while maintaining the computational efficiency of the window-based paradigm. The SKB workflow is as follows:

For the input feature map $F_{\mathrm{in}}\in R^{C\;\times \;H\;\times \;W}$, it is first processed by Layer Normalization (LN) then fed into the W-AGMSA to capture global contextual information with geometric awareness. The output is added to the original input $F_{\mathrm{in}}$ to form the first residual connection, resulting in $X^{\prime }$:

(1) $$X^{\prime }=W-AGMSA\left(\mathrm{LN}(F_{\mathrm{in}})\right)+F_{\mathrm{in}}.$$

Next, $X^{\prime }$ is normalized again and passed through the nonlinear transformation module SGR-KAN to enhance the representation of inter-pixel relationships in the image. A residual connection is applied to preserve the original features, yielding $\hat{X}$:

(2) $$\hat{X}=\mathrm{SGR-KAN}\left(\mathrm{LN}(X^{\prime })\right)+X^{\prime }.$$

The above steps are then repeated: the transformed feature $\hat{X}$ is normalized and passed through SW-AGMSA to further expand the receptive field. The result is then added to the input by a residual connection, producing $\bar{X}$:

(3) $$\bar{X}=\mathrm{SW-AGMSA}\left(\mathrm{LN}(\hat{X})\right)+\hat{X}.$$

Finally, the feature $\bar{X}$ is normalized once more and passed through the second SGR-KAN module to complete the final nonlinear fusion, resulting in the output feature map $F_{\mathrm{out}}\in R^{C\;\times \;H\;\times \;W}$:

(4) $$F_{\mathrm{out}}=\mathrm{SGR-KAN}\left(\mathrm{LN}(\bar{X})\right)+\bar{X}.$$

#### 3.2.2. Adaptive Geometry-Aware Multi-Head Self-Attention

To deeply integrate the epipolar geometry priors of stereo matching while efficiently capturing global context and long-distance dependencies in feature maps, we design the Adaptive Geometry-aware Multi-head Self-Attention (AG-MSA) module. While prior works often apply epipolar priors at cross-view or volume stages, we embed them inside single-view self-attention via AGRP. The core of the module lies in our newly proposed attention geometry bias adjustment mechanism, specifically designed for stereo matching. By consecutively applying Window-based and Shifted Window-based multi-head self-attention (W-AGMSA and SW-AGMSA) structures, the module embeds epipolar geometry priors into every stage of feature learning while maintaining computational efficiency. This guides the network to learn feature representations that are more robust to horizontal matching and less sensitive to vertical noise, thereby improving matching accuracy in globally inferred regions.

**Figure 2:**
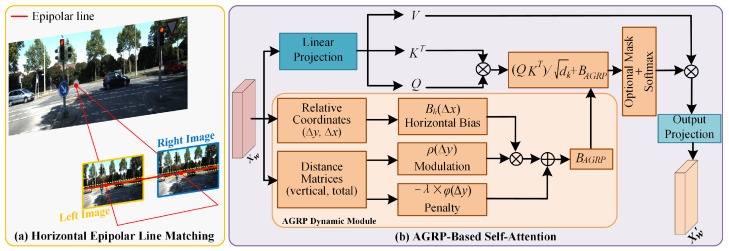
The illustration of matching along the epipolar line and the illustration of our self-attention mechanism based on AGRP.

In the standard self-attention mechanism, for a given input feature $X\in R^{C\;\times \;H\;\times \;W}$, it is first reshaped into a series of image patches (patch tokens). The standard scaled dot-product self-attention mechanism is defined as:

(5) $$\mathrm{Attention}\left(Q,K,V\right)=\mathrm{Softmax}\left(\frac{QK^{T}}{\sqrt{d_{k}}}+B\right)V,$$

where *Q*, *K*, and *V* represent the Query, Key, and Value matrices, respectively. $d_{k}$ is the dimensionality of the key vectors, and $\frac{QK^{T}}{\sqrt{d_{k}}}$ represents the attention scores. *B* denotes the relative position bias, which encodes spatial relationships. As a key factor for performance enhancement, the bias remains crucial for dense unsupervised stereo matching. However, the design of the relative position bias is inherently isotropic, treating displacements in all directions equally. As shown in Figure 2a, this fundamentally contradicts the anisotropic physical prior in rectified stereo matching, where the matching search is strictly constrained along horizontal epipolar lines.

In practical deployment, there remains a gap between ideal calibration and real-world imaging. Camera calibration errors, residual lens distortion, and slight mismatches between left and right view sensors can lead to vertical misalignment across multiple pixels. Imposing a strict zero vertical displacement constraint would compromise system robustness due to its intolerance of these inevitable perturbations. In contrast, adopting a flexible design that allows for minor vertical offsets is crucial for maintaining performance under real imaging conditions.

To address the above challenges, we propose a novel geometry bias framework based on direct geometry modeling and adaptive learning, which we refer to as Adaptive Geometry-enhanced Relative Positioning (AGRP). As shown in Figure 2b, the framework abandons indirect lookup tables and simple additive biases and instead adopts a hybrid model composed of geometric functions. The approach not only introduces strong epipolar geometric priors into the unsupervised stereo matching model but employs a distance-based dynamic constraint mechanism to overcome the issue of vertical perturbations. As a result, it facilitates the learning of disparity information that is structurally more consistent and detail-precise, particularly in challenging regions.

The approach constructs the final attention logit L(Δy,Δx) directly through two complementary geometric constraint mechanisms. Firstly, the approach encodes fine-grained spatial relationships along the epipolar line using a horizontal spatial bias $B_{h}\left(\Delta x\right)$. Secondly, a modulation factor ρ(Δy) determined by the vertical distance Δy is introduced and multiplied with $B_{h}\left(\Delta x\right)$ to dynamically adjust the contribution of the horizontal bias, thereby reducing the reliability of the horizontal bias in nonepipolar directions. Finally, beyond modulation, we introduce an independent penalty term ϕ(Δy), also determined by the vertical distance Δy, which is subtracted from the total attention logit to explicitly suppress any attention that is not horizontally aligned. The final attention logit is formulated as:

(6) $$L\left(\Delta y,\Delta x\right)=\frac{QK^{T}}{\sqrt{d_{k}}}+\left(B_{h}\left(\Delta x\right)\cdot \rho \left(\Delta y\right)\right)-\phi \left(\Delta y\right),$$

where Δx and Δy represent the relative coordinate differences in width and height directions, respectively, between two image patches (tokens) within a window. The primary horizontal bias $B_{h}\left(\Delta x\right)$ is retrieved from a learnable parameter tensor ${\hat{B}}_{h}\in R^{2M-1}$, where *M* denotes the window size.

The geometric modulation factor ρ(Δy) is controlled by a learnable, head-specific modulation strength factor $\alpha_{h}$. To reduce the reliability of spatial bias in nonepipolar directions, ρ(Δy) is designed using a stable rational function and dynamically scales the contribution of $B_{h}$ based on the vertical distance |Δy|:

(7) $$\rho \left(\Delta y\right)=\frac{1}{1+\alpha_{h}\cdot \left|\Delta y\right|}.$$

The direct geometric penalty ϕ(Δy) is controlled by a learnable, per-head penalty strength factor $\lambda_{h}$. This function is bounded and smooth, ensuring stable training while effectively imposing constraints. It is directly subtracted from the total logit to explicitly suppress any nonhorizontal alignment in attention:

(8) $$\phi \left(\Delta y\right)=\lambda_{h}\cdot \frac{\left|\Delta y\right|}{1+0.5\cdot |\Delta y|}.$$

Here, we design the geometric constraint in two complementary forms: the modulation factor ρ and the penalty term ϕ. Their synergy is key to achieving robustness. The modulation factor ρ does not directly affect the overall attention score but is specifically used to adjust the confidence of the horizontal spatial bias $B_{h}$. The underlying logic is that when a pixel deviates in the vertical direction, the predefined spatial correlation rule $B_{h}$ in the horizontal direction should accordingly lose its significance. However, relying solely on this modulation may be insufficient to suppress incorrect matches driven by extremely high content similarity $\left(QK^{T}\right)$. To this end, by combining it with the strong constraint of the direct penalty term ϕ, our AGRP framework is able to incorporate epipolar geometric prior knowledge in a manner that is both flexible and robust.

In this design, both ρ and ϕ are smooth and continuous functions of |Δy|, making the constraint effect of AGRP flexible. For minor vertical displacements caused by calibration errors (e.g., |Δy|=1), the model applies only a mild suppression. In contrast, for larger vertical displacements (e.g., |Δy|≥3), the suppression effect increases sharply. In this way, AGRP creates a soft epipolar band in the attention space, centered on the horizontal epipolar line and with probabilities smoothly decaying with vertical distance, as illustrated by the yellow dashed box in Figure 2a. This allows the model to focus on high-probability horizontal matches while tolerating small vertical perturbations that are inevitable in real-world scenarios, thereby enhancing the robustness and accuracy of matching without introducing excessive redundant context. Finally, the logarithmic values *L* adjusted by the AGRP framework are normalized via Softmax and then multiplied by the value matrix *V* to compute the attention output:

(9) $$\mathrm{Attention}\left(Q,K,V\right)=\mathrm{Softmax}\left(L(\Delta y,\Delta x)\right)V.$$

The AG-MSA module in this paper is constructed by alternately stacking W-AGMSA and SW-AGMSA layers. Within each local or shifted window, the complete AGRP computation process is executed. This enables the approach to adaptively learn feature representations that are robust to geometric structure variations at every stage of global context aggregation and information interaction.

#### 3.2.3. Spatial Group-Rational KAN

In the standard Swin Transformer, MLP with fixed activation functions and simple linear transformations limit nonlinear modeling. To address this, we draw inspiration from the core idea of the Kolmogorov–Arnold Network (KAN), which replaces MLP with flexible and learnable univariate functions. Although previous studies have explored similar approaches for specific image tasks [26,27], these approaches often suffer from poor model stability when applying KAN to more complex tasks, leading to difficulties in scaling up large-scale model training. Recently, GR-KAN [28], a novel variant of KAN, has attracted widespread attention. The variant replaces the B-spline basis with rational functions and introduces a parameter sharing mechanism across edge groups, thereby improving performance and scalability. However, GR-KAN typically requires flattening two-dimensional feature maps into one-dimensional sequences when handling visual tasks. This operation disrupts the inherent spatial structure and local topological relationships of images, resulting in the loss of relative positional information of features. This is particularly detrimental in stereo matching, where pixel-level relative positions and local structural distributions are crucial for accurate disparity estimation.

**Figure 3:**
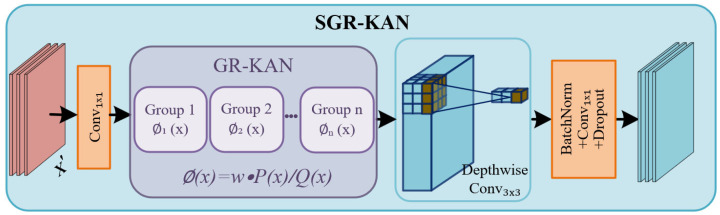
The illustration of Spatial Group-Rational KAN (SGR-KAN). It mainly consists of GR-KAN and 3 × 3 depthwise convolution.

As shown in Figure 3, to address this issue, we design a Spatial Group-Rational KAN (SGR-KAN), which preserves two-dimensional spatial structural features and serves as a powerful alternative to standard MLP. The core innovation of SGR-KAN lies in its organic integration of the powerful nonlinear modeling capability of rational function activations with the spatial locality awareness of deep convolutions. It performs channel-wise and spatial group modeling directly on 2D feature maps, thereby enhancing nonlinear expressiveness while fully preserving and leveraging critical spatial structural information.

(1) **SGR-KAN Core Activation Unit**

The core of SGR-KAN lies in its advanced activation unit, which is designed based on the theoretical foundation of Kolmogorov–Arnold Networks (KANs). The Kolmogorov–Arnold theorem [31] states that any multivariate continuous function $f(x_{1},\ldots ,x_{n})$ defined on a bounded domain can be decomposed into a weighted sum of several univariate continuous functions $\Phi_{j}$ and $\phi_{ij}$:

(10) $$f\left(x_{1},\ldots ,x_{n}\right)=\sum_{j=1}^{2n+1}\Phi_{j}\left(\sum_{i=1}^{n}\phi_{ij}\left(x_{i}\right)\right).$$

Based on this idea, the KAN architecture achieves deep mapping through the ordered nesting of *L* layer function matrices $\Phi_{i}$ (i∈{1,…,L}):

(11) $$\mathrm{KAN}\left(X\right)=\left(\Phi_{L}\circ \Phi_{L-1}\circ \cdots \circ \Phi_{2}\circ \Phi_{1}\right)X,$$

where ∘ denotes layer-wise nesting, and each layer of function matrix $\Phi_{l}\left(\cdot \right)$ consists of several learnable univariate transformations $\phi_{i,j}\left(\cdot \right)$, which are responsible for information transfer from layer *l* to layer l+1:

(12) $$\Phi_{l}\left(\cdot \right)=\left[\begin{array}{ccc} \phi_{1,1}\left(\cdot \right) & \ldots & \phi_{1,n_{l}}\left(\cdot \right)\\ \vdots & \ddots & \vdots \\ \phi_{n_{l+1},1}\left(\cdot \right) & \ldots & \phi_{n_{l+1},n_{l}}\left(\cdot \right) \end{array}\right].$$

However, directly learning the spline basis function form of ϕ(x) may face issues, such as scalability. Therefore, our SGR-KAN activation unit draws on the idea of GR-KAN and parameterizes the unary function ϕ(x) of the edge as a rational function of an *m*-order polynomial P(x) and an *n*-order polynomial Q(x). Furthermore, to address the potential instability caused by poles (i.e., denominator Q(x)→0), we adopt the Safe Padé Activation Unit (PAU) [32]:

(13) $$\phi \left(x\right)=wF\left(x\right)=\frac{wP(x)}{Q(x)}=w\cdot \frac{\sum_{i=0}^{n}a_{i}x^{i}}{1+\sum_{j=1}^{m}\left|b_{j}x^{j}\right|},$$

where $a_{i}$ and $b_{j}$ are the learnable coefficients of the rational function, and *w* is a learnable scaling factor. Taking the absolute value of the coefficients $b_{j}x^{j}$ in the denominator ensures the boundedness and numerical stability of the function.

To further improve parameter efficiency, we introduce a grouping mechanism [28] to divide the input channels into *g* groups, with each group containing $d_{g}=d_{\mathrm{in}}/g$ channels. Each channel shares the same set of learnable coefficients $a_{i}$, $b_{j}$, and *w*, where $\left\lfloor i/d_{g}\right\rfloor$ is the group index. For an input vector X, the operation can be represented as the product of a weight matrix W and a Group-Rational function vector F(X):

(14) $$\mathrm{GR-KAN}\left(X\right)=WF\left(X\right)=\left[\begin{array}{ccc} w_{1,1}\left(\cdot \right) & \ldots & w_{1,d_{\mathrm{in}}}\left(\cdot \right)\\ \vdots & \ddots & \vdots \\ w_{d_{\mathrm{out}},1}\left(\cdot \right) & \ldots & w_{d_{\mathrm{out}},d_{\mathrm{in}}}\left(\cdot \right) \end{array}\right]\left[\begin{array}{c} F_{\left\lfloor 1/d_{g}\right\rfloor }\left(x_{1}\right)\\ \vdots \\ F_{\left\lfloor d_{\mathrm{in}}/d_{g}\right\rfloor }\left(x_{d_{\mathrm{in}}}\right) \end{array}\right].$$

(2) **Full Architecture of SGR-KAN**

Based on the aforementioned activation unit, the overall structure of SGR-KAN is illustrated in Figure 3. Firstly, for an input feature $X^{\prime }\in R^{C\;\times \;H\;\times \;W}$ that has been processed by layer normalization (LayerNorm), a 1 × 1 convolution layer is applied to expand the channel dimension from *C* to $C_{\mathrm{hidden}}$, providing a richer feature space for the Group-Rational activation:

(15) $$X_{\exp}={\mathrm{Conv}}_{1\;\times \;1}\left(X^{\prime }\right).$$

Next, the expanded feature $X_{\exp}$ is fed into the GR-KAN [28] activation module to perform Group-Rational Activation:

(16) $$X_{\mathrm{act}}=\mathrm{GR-KAN}\left(X_{\exp}\right).$$

Then, a 3 × 3 Depthwise Convolution is introduced to perform spatial filtering independently on each feature channel, which effectively integrates contextual information from neighboring pixels and enhances the spatial awareness:

(17) $$X_{\mathrm{dw}}={\mathrm{DWConv}}_{3\;\times \;3}\left(X_{\mathrm{act}}\right).$$

Finally, the channel dimension is projected back from $C_{\mathrm{hidden}}$ to the original channel dimension *C* through another 1 × 1 convolution layer:

(18) $$\mathrm{SGR-KAN}\left(X^{\prime }\right)={\mathrm{Conv}}_{1\;\times \;1}\left(X_{\mathrm{dw}}\right).$$

Therefore, the proposed SGR-KAN combines learnable rational activation functions with convolution operations that preserve spatial topology, not only retaining the powerful nonlinear expressive capability of KAN but overcoming its inherent limitations when handling structured data including images. Furthermore, the grouping mechanism ensures its parameter efficiency.

### 3.3. Dynamic Candidate Gated Fusion

In unsupervised stereo matching, accurately capturing the geometric structure of complex scenes and ill-posed regions requires effectively fusing CNN features rich in local details with Stereo-KANformer features that provide geometry-aware global context. To address key challenges arising from the fusion of heterogeneous features, such as information redundancy, detail loss, and gradient conflicts, we propose a novel Dynamic Candidate Gated Fusion (DCGF) module. This module abandons the fixed, single-path update paradigm of traditional fusion approaches and introduces a fine-grained fusion architecture composed of three core stages: spatial pre-enhancement, dynamic parallel candidate construction, and adaptive fusion and update.

First, in the spatial pre-enhancement stage, Coordinate Attention [33] is utilized to pre-enhance the directional awareness and long-distance spatial dependencies of the concatenated heterogeneous features, achieving preliminary alignment and information enhancement of global and local features at the spatial context level. Second, in the dynamic parallel candidate construction stage, traditional gated fusion approaches typically generate only a fixed candidate state [34]. To improve the flexibility and expressive capability of the fusion process, we propose a Dynamic Dual-Candidate State mechanism. This mechanism employs separated dual reset gates to independently and finely control the local and global information flows and then generates two complementary update schemes in parallel: a primary candidate state, representing a stable combination of features; and an enhanced candidate state, which is specifically designed to capture deeper and more complex synergistic relationships between the two types of features through nonlinear operations including cross-modulation. Finally, during the adaptive fusion and update phase, the module employs a lightweight channel attention submodule to dynamically learn the fusion weights based on the content of the two candidate states. The optimally weighted candidate state is then fused with the original local features under the control of the update gate. The workflow of the DCGF module is as follows:

(1) **Attention Enhancement and Gating Signal Generation**

First, the input local feature $F_{\mathrm{local}}\in R^{C\;\times \;H\;\times \;W}$ and global feature $F_{\mathrm{global}}\in R^{C\;\times \;H\;\times \;W}$ are concatenated along the channel dimension. To enhance the spatial awareness of the features, we apply a coordinate attention module [33] to the concatenated features to capture direction-aware and position-sensitive global contextual information and cross-region disparity consistency, resulting in the enhanced feature $F_{\mathrm{att}}$.

Then, based on this, we generate the current step *t* gating signals using three independent 1 × 1 convolutions followed by a Sigmoid activation function σ, including the update gate $Z_{t}$, the local reset gate $R_{\mathrm{local},t}$, and the global reset gate $R_{\mathrm{global},t}$:

(19) $$\left\{\begin{array}{c} Z_{t}=\sigma \left({\mathrm{Conv}}_{z}\left(F_{\mathrm{att}}\right)\right),\\ R_{\mathrm{local},t}=\sigma \left({\mathrm{Conv}}_{r,\mathrm{local}}\left(F_{\mathrm{att}}\right)\right),\\ R_{\mathrm{global},t}=\sigma \left({\mathrm{Conv}}_{r,\mathrm{global}}\left(F_{\mathrm{att}}\right)\right). \end{array}\right.$$

(2) **Dynamic Parallel Dual Candidate State Construction**

To provide richer fusion possibilities, we design two parallel candidate states. First, dual reset gates are applied to the original features $F_{\mathrm{local}}$ and $F_{\mathrm{global}}$ respectively, through element-wise multiplication ⊙ to selectively reset them:

(20) $$\left\{\begin{array}{c} F_{\mathrm{local}}^{\prime }=R_{\mathrm{local},t}⊙F_{\mathrm{local}},\\ F_{\mathrm{global}}^{\prime }=R_{\mathrm{global},t}⊙F_{\mathrm{global}}. \end{array}\right.$$

Subsequently, based on the reset concatenated features, a main candidate state ${\tilde{H}}_{\mathrm{main},t}$ is constructed using a 1 × 1 convolution followed by a tanh activation function:

(21) $${\tilde{H}}_{\mathrm{main},t}=\tanh\left({\mathrm{Conv}}_{h}\left(\mathrm{Concat}\left(F_{\mathrm{local}}^{\prime },F_{\mathrm{global}}^{\prime }\right)\right)\right).$$

To capture deeper nonlinear interactions between the two features, the enhanced candidate state ${\tilde{H}}_{\mathrm{enhanced},t}$ is constructed through cross modulation, which involves element-wise multiplication and addition of the reset features:

(22) $$F_{\mathrm{cross}}=\mathrm{Concat}\left(F_{\mathrm{local}}^{\prime }⊙F_{\mathrm{global}}^{\prime },\;F_{\mathrm{local}}^{\prime }+F_{\mathrm{global}}^{\prime }\right).$$

The enhanced candidate state is then activated using a 1 × 1 convolution followed by a tanh activation function:

(23) $${\tilde{H}}_{\mathrm{enhanced},t}=\tanh\left({\mathrm{Conv}}_{h,\mathrm{enh}}\left(F_{\mathrm{cross}}\right)\right).$$

(3) **Adaptive Fusion and Update**

First, to achieve adaptive combination of the two candidate states, a lightweight channel attention submodule SubAtt is introduced, which is implemented using a 1 × 1 convolution and global average pooling (GAP). The module takes the concatenated candidate states ${\tilde{H}}_{\mathrm{concat}}$ as input and outputs the normalized fusion weights $W_{\mathrm{main}}$ and $W_{\mathrm{enhanced}}$:

(24) $$\mathrm{SubAtt}\left({\tilde{H}}_{\mathrm{concat}}\right)={\mathrm{Conv}}_{1\;\times \;1}\left(\mathrm{ReLU}\left({\mathrm{Conv}}_{1\;\times \;1}\left(\mathrm{GAP}\left({\tilde{H}}_{\mathrm{concat}}\right)\right)\right)\right),$$

(25) $$\left[W_{\mathrm{main}},W_{\mathrm{enhanced}}\right]=\mathrm{Softmax}\left(\mathrm{SubAtt}({\tilde{H}}_{\mathrm{concat}})\right).$$

Next, the optimal candidate state ${\tilde{H}}_{t}$ is obtained through weighted fusion:

(26) $${\tilde{H}}_{t}=W_{\mathrm{main}}⊙{\tilde{H}}_{\mathrm{main},t}+W_{\mathrm{enhanced}}⊙{\tilde{H}}_{\mathrm{enhanced},t}.$$

Finally, under the control of the update gate $Z_{t}$, the optimal candidate state is combined with the local feature to produce the fused feature $F_{\mathrm{final}}$:

(27) $$F_{\mathrm{final}}=\left(1-Z_{t}\right)⊙F_{\mathrm{local}}+Z_{t}⊙{\tilde{H}}_{t}.$$

The DCGF dynamically adjusts the degree of local detail preservation and the proportion of global context incorporation based on the characteristics of the input features. This effectively overcomes the shortcomings of traditional fusion strategies and yields more discriminative feature representations, thereby enhancing the fusion capability of global and local information, especially in complex regions.

### 3.4. Adaptive Geometry-Aware Stereo-KANformer Network

The proposed Adaptive Geometry-aware Stereo-KANformer Network (AGSK-Net) is constructed by systematically integrating the designed modules, namely AG-MSA, SGR-KAN, and DCGF, into a unified architecture. This section focuses on how they are organized into the final network and how key parameters are configured. $C_{0}$, $C_{1}$, …, $C_{4}$ denote feature maps at different hierarchical stages of the network, where deeper levels correspond to lower spatial resolutions and larger channel dimensions. The overall pipeline is illustrated in Figure 1a.

**Shallow stages (1, 1/2 resolutions):** Standard convolutional layers are applied to preserve high-resolution local structures while maintaining computational efficiency.

**Deep stages (1/4, 1/8, 1/16 resolutions):** At these scales, Stereo-KANformer Blocks (SKBs) are employed. Each SKB integrates AG-MSA and SGR-KAN, where the number of attention heads is set to 8. Hierarchical window sizes are assigned as 8 × 8 and 16 × 16 with corresponding shifted windows of 4 × 4 and 8 × 8, respectively, enabling efficient cross-window information exchange. The number of SKBs per stage is set to 1, 1, 2 for progressively deeper modeling.

**Fusion across scales:** DCGF modules are deployed after each SKB stage to merge high-level global features with low-level local details. By dynamically adjusting the fusion weights through candidate gating, these modules ensure consistent feature refinement across different resolutions.

Through this hierarchical integration, AGSK-Net combines fine-grained local cues, geometry-aware global reasoning, and adaptive multi-scale fusion, delivering high-quality disparity representations with improved robustness in ill-posed regions.

## 4. Experiment and Analysis

### 4.1. Experimental Data and Environment

**KITTI 2012/2015.** The KITTI 2012 dataset contains 194 pairs of training images and 195 pairs of testing images, all with a resolution of 376 × 1240. The KITTI 2015 dataset includes 200 pairs of dynamic street scene training images and 200 pairs of testing images, also with a resolution of 376 × 1240.

**Scene Flow.** Scene Flow is a synthetic dataset composed of three subsets: Flyingthings3D, Driving, and Monkaa, comprising a total of 35454 pairs of training images and 4370 pairs of testing images, with a resolution of 960 × 540.

**Middlebury 2021.** Middlebury 2021 is a real indoor scene dataset captured using structured light, containing 24 pairs of high-resolution images.

**Experiment details.** The approach in this study is implemented based on Pytorch 1.10 and Python 3.10. The hardware configuration includes an Intel Core i7-11700F 2.50 GHz processor, 32 GB of RAM, and two NVIDIA RTX 4060TI graphics cards with 16 GB of VRAM each. To enable comparison with the baseline, the hyperparameters in this paper are set to be consistent with PASMNet. The batch size is set to 8, and the optimizer used is Adam, with $\beta_{1}=0.9$ and $\beta_{2}=0.999$. To ensure fair and comprehensive evaluation, we adopted a multi-stage training and testing strategy on four benchmark datasets: Scene Flow, KITTI 2012, KITTI 2015, and Middlebury 2021. The model was first pre-trained on Scene Flow (35,454 training pairs, 4370 test pairs) for 15 epochs, with the learning rate set to $1\;\times \;10^{-3}$ for the first 10 epochs and $1\;\times \;10^{-4}$ for the last 5 epochs. This stage provides dense disparity supervision under diverse synthetic conditions. We then fine-tuned on KITTI 2015 (200 pairs) and KITTI 2012 (194 pairs) for 100 epochs, using a learning rate of $1\;\times \;10^{-4}$ for the first 80 epochs and $1\;\times \;10^{-5}$ for the last 20, to adapt the model to real driving scenes. For evaluation, we tested on the Scene Flow (4370 pairs), KITTI 2012 (195 pairs), and KITTI 2015 (200 pairs) test sets and directly tested on Middlebury 2021 (24 high-resolution pairs) without adaptation to assess detail recovery, robustness in complex regions, and cross-dataset generalization.

### 4.2. Ablation Study of AGSK-Net

To systematically analyze the impact of each component in the AGSK-Net model on unsupervised stereo matching performance, multiple ablation experiments were conducted on the Scene Flow and KITTI 2015 datasets in Table 1, with different experimental configurations distinguished by index labels IDi (i = 1, 2, 3, 4, 5).

#### 4.2.1. Effect on Swin Transformer

In this experiment, the classic Swin Transformer was first integrated to enhance the feature extraction capability for capturing global context. Comparing the results of ID1 and ID2 (ID1 + Swin Transformer) in Table 1, the EPE and 3-pixel error are decreased by 7.78% and 5.48%, respectively. Compared to using only a CNN model, the Swin Transformer enhances the global inference capability by leveraging the window-based and shifted window multi-head attention mechanisms to extract global context information.

#### 4.2.2. Effect on Stereo-KANformer

This paper proposes an improved Stereo-KANformer by incorporating AG-MSA and SGR-KAN to introduce epipolar geometric priors and further enhance nonlinear expressive capability in complex regions. According to the comparison between ID2 and ID3 (ID2 + AGRP bias adjustment mechanism) in Table 1, the EPE and 3-pixel error are reduced by 7.36% and 4.48%, respectively. From the comparison between ID3 and ID4 (ID3 + SGR-KAN), the EPE and 3-pixel error are reduced by 19.16% and 9.10%, respectively. This demonstrates that the proposed AG-MSA introduces geometric priors into the attention mechanism to obtain geometry-aware global contextual information and that SGR-KAN effectively enhances the nonlinear expressive capability in complex regions through the Spatial Group-Rational KAN architecture.

#### 4.2.3. Effect on DCGF

The proposed DCGF adaptively fuses global and local information at multiple scales to extract rich global context and local detail features. The comparison between ID4 and ID5 (ID4+DCGF) in Table 1 shows that after introducing the DCGF adaptive fusion mechanism, EPE and 3-pixel error are reduced by 23.70% and 6.70%, respectively. This demonstrates that DCGF effectively integrates contextual and detailed information at all scales through coordinate attention enhancement and a multi-candidate dynamic gating mechanism, thereby improving the accuracy of unsupervised stereo matching.

#### 4.2.4. Detailed Analysis of AGRP and Network Architecture

To further investigate the specific design choices within our framework, we conducted a series of more detailed ablation studies, with results presented in Table 2.

**Analysis of AGRP Components:** To validate our hybrid geometric bias design in AG-MSA, we analyzed the individual contributions of the geometric modulation (ρ) and the direct penalty (ϕ) terms. We started with the Swin Transformer baseline (ID2) and added each component separately. The “M-only” variant (ID3a), which only uses the modulation factor ρ to scale the horizontal bias, already shows a significant performance improvement, confirming the benefit of down-weighting the spatial prior for off-epipolar pixels. The “P-only” variant (ID3b), which only applies the direct penalty ϕ, yields an even stronger improvement, highlighting the effectiveness of an explicit, absolute penalty. Our full AGRP model (ID3), which synergistically combines both mechanisms, achieves the best performance. This demonstrates that the two components are complementary: the modulation provides a nuanced, relative adjustment of the spatial bias, while the penalty acts as a robust, absolute deterrent against geometrically inconsistent matches.

**Analysis of Multi-Scale SKB Architecture:** To justify the necessity of our multi-scale design, we compared the full model with three variants. ID4a, which places Stereo-KANformer Blocks (SKBs) only at the deepest 1/16 layer, shows limited performance improvement, likely because global inference information can be effectively extracted only at low resolution. ID4b, which applies SKBs at both the 1/16 and 1/8 resolution levels, performs better, indicating that incorporating higher-resolution SKBs allows simultaneous extraction of both global and local information. Finally, the superior performance of ID4c demonstrates that hierarchically applying SKBs across multiple resolutions is crucial for effectively capturing and integrating fine details with abstract global context.

**Analysis of Stereo-KANformer Depth:** Finally, we analyzed the impact of the number of SKB blocks (i.e., the depth of the transformer backbone). We compared our final configuration “Medium Depth” (ID4e) with a “Shallow” variant (ID4d) that uses fewer SKB blocks and a “Deep” variant (ID4f) that uses more. The “Shallow” model slightly improves the performance, indicating insufficient feature transformation capacity. The “Deep” model yields a marginal improvement on the synthetic Scene Flow dataset but a slight gain on the real-world KITTI dataset, suggesting a potential for overfitting and a less favorable performance-complexity trade-off. These results validate that our chosen depth provides a well-balanced and effective configuration for the task.

### 4.3. Benchmark Evaluation

The proposed Adaptive Geometry-aware Stereo-KANformer Network (AGSK-Net) aims to enhance the accuracy and generalization performance of unsupervised stereo matching. The model is first trained on the Scene Flow dataset and then fine-tuned separately on the KITTI 2012 and KITTI 2015 training sets.

#### 4.3.1. Quantitative Evaluation

To ensure a fair and objective comparison against prior state-of-the-art methods, our quantitative evaluation relies exclusively on the most widely adopted and standardized metrics in the stereo matching field: the End-Point Error (EPE) and the N-pixel error rate. These metrics are universally used by community benchmarks and allow for direct, apples-to-apples performance assessment. Table 3 presents the testing results of our approach on the KITTI 2015 and Scene Flow benchmarks. In the table, “D1-bg”, “D1-fg”, and “D1-all” correspond to the 3-pixel error metric [35,36] for background pixels, foreground pixels, and all-region pixels, respectively. As shown in Table 3, the proposed approach achieves competitive performance across all regions of the KITTI 2015 and Scene Flow datasets compared to other representative state-of-the-art approaches, further demonstrating the effectiveness of AGSK-Net.

In addition, Table 4 presents the results on the KITTI 2012 test set. It can be observed that the proposed approach achieves the lowest error across multiple metrics, such as 2, 3, and 5-pixel errors [37]. AGSK-Net significantly outperforms PASMNet [12] in terms of accuracy by extracting and adaptively fusing multi-scale global context and local details through Stereo-KANformer and DCGF.

**Table 3:** The results on the KITTI 2015 benchmark. “All” refers to the entire image region, “Noc.” refers to the nonoccluded regions in the image. The “-” symbol indicates that the result is not found in the original paper.

Models	KITTI 2015						Scene Flow
	All/%			Noc./%			EPE/Pixel
	D1-bg	D1-fg	D1-All	D1-bg	D1-fg	D1-All	
3DG-DVO [38]	14.12	18.68	14.88	13.54	17.27	14.16	-
Zhou [10]	-	-	10.23	-	-	9.91	-
OASM-Net [39]	6.89	19.42	8.98	5.44	17.30	7.39	3.86
SegStereo [40]	-	-	8.79	-	-	7.70	-
Self-SuperFlow [41]	5.78	19.76	8.11	4.69	18.29	6.93	-
AAFS [42]	6.27	13.95	7.54	5.96	13.01	7.12	2.88
PASMNet [12]	5.41	16.36	7.23	5.02	15.16	6.69	3.54
Permutation Stereo [43]	5.53	15.47	7.18	5.18	14.51	6.72	-
SPSMnet [44]	5.42	12.84	6.65	4.94	12.01	6.10	-
UHP [45]	5.00	13.70	6.45	4.65	12.37	5.93	-
CRD-Fusion [46]	4.59	13.68	6.11	4.30	12.73	5.69	-
Ours	**4.44**	**12.40**	**5.69**	**4.36**	**10.36**	**5.68**	**2.64**

**Table 4:** The results on the KITTI 2012 benchmark. We present the evaluated errors at thresholds of 2-pixel, 3-pixel, and 5-pixel errors on the benchmark.

Models	>2-Pixel/%		>3-Pixel/%		>5-Pixel/%	
	Noc.	All	Noc.	All	Noc.	All
Zhou [10]	-	14.32	-	9.86	-	7.88
SegStereo [40]	-	-	7.89	9.64	-	-
OASM-Net [39]	9.01	11.17	6.39	8.60	4.32	6.50
Permutation Stereo [43]	11.89	13.16	7.39	8.48	4.32	5.11
PASMNet [12]	8.77	10.58	5.91	6.98	3.86	4.67
UHP [45]	9.08	10.37	6.05	7.09	3.69	4.43
AAFS [42]	10.64	11.69	6.10	6.94	3.28	3.81
Ours	**7.24**	**8.85**	**4.84**	**5.74**	**3.08**	**3.71**

#### 4.3.2. Qualitative Evaluation

**Figure 4:**
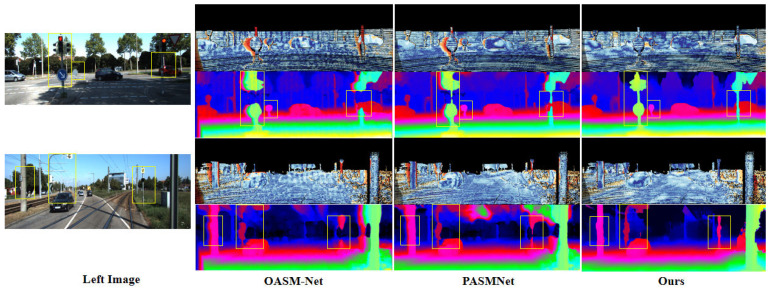
Predicted disparity maps on the KITTI 2015 test dataset. Our approach achieves better performance than PASMNet and OASM-Net in complex regions, such as occlusions, weak textures, and small objects, etc.

**Figure 5:**
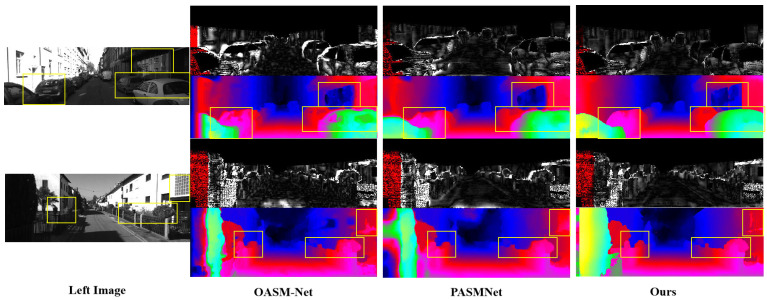
Predicted disparity maps on the KITTI 2012 test dataset. The results of our approach perform better than PASMNet and OASM-Net in ill-posed regions, such as occlusions, repetitive textures, and edges, etc.

Figure 4 and Figure 5, respectively, illustrate the error maps and depth maps generated by our approach and the baseline PASMNet [12] and OASM-Net [39] on the KITTI 2015 and KITTI 2012 test sets.

Compared with PASMNet and OASM-Net, our approach leverages Stereo-KANformer to capture multi-scale global context and local detail information and adaptively fuses them through DCGF, resulting in significantly better overall performance. In particular, the yellow rectangles in Figure 4 highlight the ill-posed regions, including object geometric structures, traffic lights, edge details, and occlusions, where our approach achieves more accurate disparity estimation.

Figure 5 further demonstrates issues such as disparity detail loss caused by fine objects, occlusions, weak textures, and object edges, which are especially common in stereo matching. As shown in the yellow rectangle, compared with other approaches, the proposed approach demonstrates more robust performance in complex ill-posed regions, such as the fine wooden frames along glass edges, repetitive textures on cars, and object boundaries and intricate fence details.

**Figure 6:**
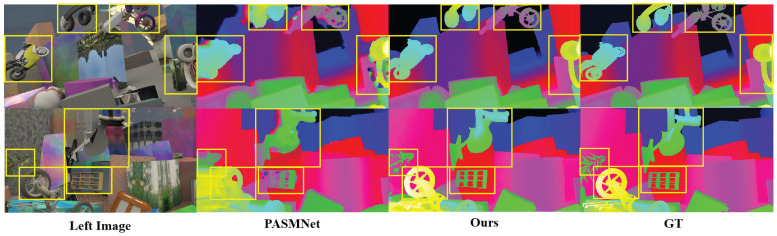
Predicted disparity maps on the Scene Flow test set. The results of our approach perform better than PASMNet in regions with occlusions, edges, and complex geometric details, etc. The GT represents the ground-truth disparity.

Furthermore, Figure 6 presents a comparison of disparity estimation performance between the proposed approach and the baseline PASMNet on the Scene Flow test set. By comparing the areas marked with yellow rectangular boxes, it is evident that the proposed approach outperforms the baseline in challenging and fine-grained regions, such as occluded cylinders, wheels, motorcycles, and headphones. This further validates the effectiveness of the proposed AGSK-Net with geometric perception capability in jointly modeling multi-scale global information and local geometric details.

### 4.4. Generalization Performance

In unsupervised deep stereo matching, generalization ability plays a key role. Therefore, we evaluate the generalization performance of our approach against the baseline PASMNet on the Middlebury 2021 dataset. For fairness, all models are exclusively pre-trained on the Scene Flow dataset without any fine-tuning on Middlebury 2021.

**Figure 7:**
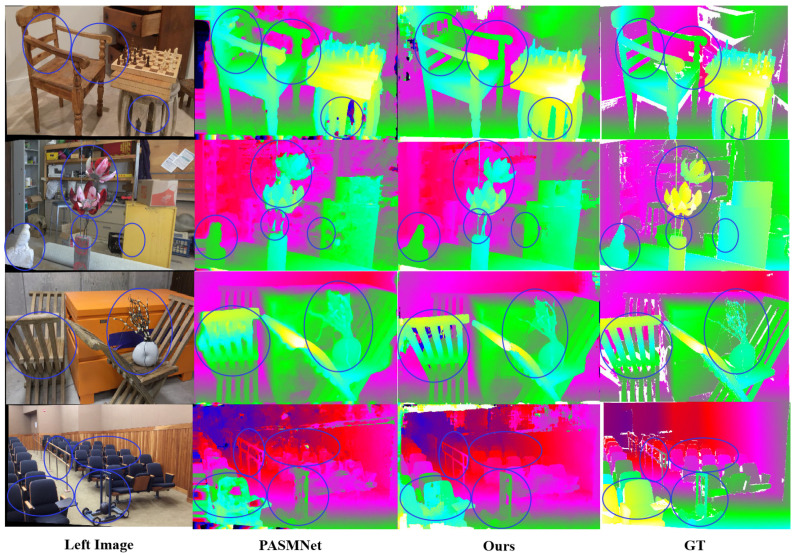
Predicted disparity maps on the Middlebury 2021 set. The results of our approach perform better than PASMNet in regions with occlusions, edges, and complex geometric details, etc.

Figure 7 presents the qualitative evaluation results on Middlebury 2021. It can be observed that our approach outperforms the baseline PASMNet in the areas marked by blue circles. Our approach demonstrates excellent generalization in disparity estimation in complex ill-posed regions, such as low-texture areas, fine details, and occlusions. For example, in the second column of Figure 7, the intricate details of the lotus leaf model, as well as the chair backs in the first, third, and fourth columns, show large disparity gaps in the results from the baseline, whereas our approach produces more complete and accurate disparity estimations. The quantitative results in Table 5 further support this conclusion. Compared with the baseline model, our approach reduces the 3-pixel error and EPE on the Middlebury 2021 dataset by 29.25% and 31.80%, respectively. Overall, by leveraging the geometry-aware global context extraction of Stereo-KANformer and the multi-scale global and local detail fusion of DCGF, our approach demonstrates significantly better generalization performance in unseen scenarios than the baseline PASMNet.

## 5. Conclusions

This paper addresses the core challenges in unsupervised stereo matching within existing deep learning paradigms, including the lack of global contextual information guided by geometric priors, limitations in modeling nonlinear relationships, and insufficient multi-scale feature fusion. We propose a novel Adaptive Geometry-aware Stereo-KANformer Network (AGSK-Net). Through the collaborative operation of multiple innovative modules, the network overcomes the limitations of traditional unsupervised stereo matching algorithms, such as the lack of global inference and geometric perception capabilities, restricted nonlinear expressive in complex regions, and inadequate fusion of global and local information. These improvements enhance matching accuracy and robustness in complex ill-posed regions. Experimental results fully validate the effectiveness of AGSK-Net and demonstrate the critical role of each proposed module in improving the performance of unsupervised stereo matching. Although AGSK-Net has made significant progress, there are still several directions worth exploring. For example, while the learnable rational functions in SGR-KAN offer strong expressive capability, they incur higher computational costs compared to standard MLP. Future work could investigate more lightweight implementations of KAN or model distillation techniques. In addition, this paper primarily focuses on binocular static images, and extending the geometric perception capability of AG-MSA to the temporal dimension and applying AG-MSA to multi-view stereo (MVS) or stereo video matching tasks represents a highly promising research direction. We believe that the proposed approach of deeply integrating domain prior knowledge into the attention mechanism will provide valuable insights for future research in 3D computer vision.

## Figures and Tables

**Table 1:** The ablation study of AGSK-Net. We evaluate EPE on the Scene Flow test dataset and the percentage of 3-pixel error on the KITTI 2015 evaluation dataset.

ID	AGSK-Net Setting				Scene Flow	KITTI 2015
	Swin Transformer	Stereo-KANformer		DCGF	EPE/Pixel	>3-Pixel/%
		AG-MSA	SGR-KAN			
ID1					5.01	7.438
ID2	✓				4.62	7.030
ID3		✓			4.28	6.715
ID4		✓	✓		3.46	6.104
ID5		✓	✓	✓	**2.64 **	**5.695 **

**Table 2:** Detailed ablation studies are conducted on the components of AGRP and the overall architecture of AGSK-Net. M-only denotes the use of only the Modulation term (ρ) in AGRP, while P-only denotes the use of only the Penalty term (ϕ). Single-Scale (1/16) applies SKB solely at the 1/16 resolution. Multi-Scale (1/16, 1/8) applies SKBs at the 1/16 and 1/8 resolutions. Multi-Scale (Fine, 1/16, 1/8, 1/4) applies SKBs at the 1/16, 1/8, and 1/4 resolutions. Shallow, Medium Depth, and Deep refer to reducing or increasing the number of SKB blocks, respectively.

ID	Ablation Component	Setting	Scene Flow EPE/Pixel	KITTI 2015 >3-Pixel/%
ID2	Baseline	Swin-Transformer	4.62	7.030
ID3a	AGRP Components	M-only (ρ)	4.45	6.881
ID3b		P-only (ϕ)	4.36	6.802
ID3		Modulation + Penalty	4.28	6.715
ID4a	SKB Architecture	Single-Scale (1/16)	2.98	6.313
ID4b		Multi-Scale (1/16, 1/8)	2.90	6.265
ID4c		Multi-Scale (1/16, 1/8, 1/4)	2.82	6.104
ID4d	SKB Depth	Shallow (1,1,1)	2.82	6.104
ID4e		Medium Depth (1,1,2)	2.64	5.695
ID4f		Deep (1,2,2)	2.63	5.693

**Table 5:** The evaluation on Middlebury 2021. We evaluate the generalization performance by percentage of 3-pixel error and EPE.

Models	Middlebury 2021	
	>3-Pixel/%	EPE/Pixel
PASMNet [12]	25.568	9.854
Ours	**18.089**	**6.720**

## Data Availability

The original contributions presented in this study are included in the article. Further inquiries can be directed to the corresponding authors.
